# COVID-19 Co-Infection May Promote Development of Sinusitis Complication in Children

**DOI:** 10.3390/children9111636

**Published:** 2022-10-27

**Authors:** Anna K. Szewczyk, Krystyna Mitosek-Szewczyk

**Affiliations:** 1 Doctoral School, Medical University of Lublin, 7 Chodźki Street, 20-093 Lublin, Poland; 2 Department of Neurology, Medical University of Lublin, 7 Chodźki Street, 20-093 Lublin, Poland; 3 Department of Child Neurology, Medical University of Lublin, 7 Chodźki Street, 20-093 Lublin, Poland

**Keywords:** children, COVID-19, SARS-CoV-2, neurological manifestations, sinusitis, complications, abscess, thrombotic complications

## Abstract

Background: The olfactory dysfunction that occurs during a COVID-19 infection has sparked much debate about its similarity to sinusitis. Up to 65% of COVID-19 pediatric patients may be asymptomatic; however, when symptoms are observed, fever and cough are the most common. Nasal congestion and discharge as well as headaches can also be seen, which makes both entities, i.e., COVID-19 and sinusitis, similar to each other. Methods: In this review, we present the clinical case of a teenager with a history of acute sinusitis and COVID-19 co-infection followed by purulent meningoencephalitis. We aim to summarize available findings on the association between COVID-19, sinusitis, and possible common complications of both diseases. Results: Differentiating between COVID-19 and sinusitis can be confusing because presented symptoms may overlap or mimic each other. Increased risk of complications, especially in patients with bacterial sinusitis co-infected with SARS-CoV-2, should prompt physicians to monitor young patients and inform parents about disturbing symptoms and possible complications. Conclusions: Acute sinusitis and COVID-19 co-infection may lead to numerous complications and should be included among the factors predisposing to worse prognosis. It is especially related to patients with high risk factors and even more important in children as they often pass the infection asymptomatically and its complications can lead to loss of health or life.

## 1. Introduction

In both pediatric and adult populations, sinusitis is a common complaint. Rhinosinusitis is more precise since symptoms of rhinitis are almost always observed; however, the authors decided to use the term “sinusitis”. Usually, this disease is characterized by inflammation of the sinus and nasal mucosa. Every year, children have on average 3–8 viral infections, which increases the possibility of developing an acute bacterial sinusitis. A vicious circle can arise as both types lead to mucosal edema and the production and retention of mucus, as well as stasis of secretion, obstruction of the ostia, impaired mucociliary clearance, and reduced aeration of the sinuses. As predisposing factors, local and systemic factors can be mentioned. The first group includes, upper respiratory infections, anatomic abnormality, nasal polyps, and tumor, while the second contains immune deficiency, cystic fibrosis, ciliary disorder, and Wegener’s granulomatosis [1,2].

In children between five and eighteen years of age, there is a relatively substantial risk of severe sinusitis complications. Nevertheless, these symptoms are often nonspecific and diagnostically difficult. A prominent level of vigilance and a complete evaluation of the patient seems crucial in the youth population. To assess the need of surgical procedure, imaging tests have a critical role [3]; however, recommendations stated that imaging studies should be performed in case of suspected complications, failure to respond to appropriate treatment, or recurrent bacterial sinusitis [4,5]. 

Although post-infectious sinusitis and chronic sinusitis (CS) are (the most common) part of the olfactory dysfunction, it was the severe acute respiratory syndrome coronavirus 2 (SARS-CoV-2, known also as COVID-19) pandemic that brought the problem to light. Olfactory dysfunction occurs in 56–78% of patients with CS, while its frequency in COVID-19 infection is reported to be between 5 and 85%, depending on the country of study. A potential protective role against SARS-CoV-2 infection was observed in CS with nasal polyps endotype 2 patients (this includes, for example, asthma and other allergic rhinitis), which is explained by the decreased expression of the angiotensin-converting enzyme 2 (ACE2) receptors in sinonasal epithelial cells (eosinophilic type characterized by high levels of activated TH 2 cell-released cytokines, innate lymphoid cell 2, and infiltrating eosinophils and mast cells) [6,7]. Available data report similar frequency of SARS-CoV-2 infection in the general population and in CS patients, which allows to exclude CS from COVID-19 risk factors [8]. The second type, acute sinusitis, is a symptomatic inflammation caused by microorganisms (primarily viruses), and affects approximately 1% of children each year. Patients with this infection normally recover within 2 weeks without any treatment. Moreover, between 0.5 and 2% of these patients will develop acute bacterial sinusitis [9,10]. In the absence of a response to proper treatment, humoral immune deficiencies are probable. This specific antibody deficiency—SAD (primary B cell immunodeficiency) is more common in adults than children, occurring in 11.9% and 6.4% of the populations, respectively. SAD was found in 11% of children with recurrent respiratory infections, whereas this immunodeficiency is often transient and may be resolved unconstrained [11,12].

In this review, we will present the case of a teenager with a history of acute sinusitis and COVID-19 co-infection followed by purulent meningoencephalitis. We aimed to summarize available findings on the association between COVID-19 infection, sinusitis, and possible common complications of both diseases.

To identify the relevant literature, we researched the electronic databases PubMed and MEDLINE for articles published in English, French, or Polish. Titles and abstracts of publications were searched with the following keywords: children, pediatric, COVID-19, SARS-CoV-2, viral respiratory tract infection, sinusitis, sinus inflammation, encephalitis, neurological manifestations, neurological complications, brain abscesses, pulmonary abscess, and thrombosis. The search, based on the titles and abstracts of all reports identified through electronic databases, was conducted by both authors independently to identify the studies matching the assumed clinical problem. In order to supplement the literature, a manual search was also carried out. In case of uncertainty, the full text of the article was obtained and discussed. Pediatric cases (<18 years old) were mainly searched; however, if the assumed results for this population were not found, the search was extended to include literature on adults.

## 2. Case Report

**Figure 1:**
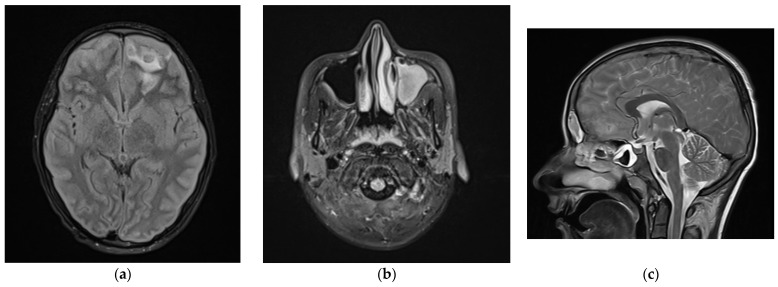
Magnetic resonance imaging (MRI) of the head of 10 December 2021 revealed: (**a**) in the left frontal lobe several irregular fluid areas features of intracerebral abscesses with mass effect; (**b**) massive inflammation of the left maxillary sinus; (**c**) sagittal sinus thrombosis.

In early December 2021, a 15-year-old male patient was transferred from the district hospital to the University Children’s Hospital in Lublin, Poland, due to the consciousness disturbances. Erenow, the boy complained of catarrh and nasal obstruction and periodic headaches that lasted several days; however, he was not taking any medications. The medical history was insignificant. In the emergency department, performance of a rapid antigen test for COVID-19 ruled out fresh infection. The patient was not vaccinated against COVID-19. At the admission, the patient’s general state was serious with Glasgow Coma Scale (GCS) score of 10 points (1 + 4 + 5) and vital signs within normal limits. Positive meningeal signs and no pyramidal symptoms were observed. Magnetic resonance imaging (MRI) of the head revealed in the left frontal lobe several irregular fluid areas features of intracerebral abscesses with mass effect and contrast enhancement of the meninges in the frontal and parietal areas with a predominance of the left side. Additionally, sagittal thrombosis, as well as massive inflammation (near complete opacification) of the left maxillary, both frontal, the ethmoid, and the sphenoid sinuses were also shown (Figure 1a–c). Computed tomography (CT) of the chest detected several confluent abscesses located in the right lung without contrast enhancement, as well as bilateral atelectasis, which might correspond to complicated pneumonia. 

The results of additional examinations revealed positive SARS-CoV-2 antibodies in type M and G immunoglobulins (IgM and IgG) classes (test was performed using the CLIA method on the Diasorin Liaison apparatus): anti-SARS-CoV-2 IgM—3.38 INDEX (test result negative: INDEX < 1.1) and SARS-CoV-2 Spike (Trimer) IgG—292.00 BAU/mL (test result negative: <33.8 BAU/mL). The lumbar puncture was performed with results indicative of inflammation (protein level 118 mg/dL, glucose 82 mg/dL, cytosis 65 cells/uL, neutrophils 76%). Among the results of the laboratory tests, positive inflammatory exponents like C-reactive protein 27.82 mg/dL (norms between 0 and 0.5 mg/dL) with procalcitonin level at 1.71 ng/mL (norms < 0.5 ng/dL) were obtained, despite negative urine and blood culture. The boy underwent laryngological and neurosurgical consultations and the broad-spectrum antibiotic therapy maintenance (third-generation cephalosporin and vancomycin) recommendations, as well as initiation of treatment with intravenous glucocorticosteroids. During observation, the patient received antibiotic therapy according to the recommendations of The Infection Prevention and Control Team, including third-generation cephalosporin and vancomycin for 9 days supplemented with β-lactam antibiotic for 7 days, then third-generation cephalosporin, vancomycin and metronidazole, with the recommendation to use for not less than 4–8 weeks (treatment of CNS and lung abscesses) under the effect control in imaging tests. After neurosurgery re-consultation, antibiotic therapy was changed to trimethoprim/sulfamethoxazole and linezolid. During treatment, this patient also received immunoglobulins with steroid therapy, intravenous mannitol with loop diuretics, as well as anticoagulation therapy with low molecular weight heparin (LMWH) in therapeutic dosage. The short-term desired effect of improving the general and neurological condition was obtained. The patient was neurologically stable, with a GCS score of 14/15, and the respiratory and circulatory systems were efficient.

**Figure 2:**
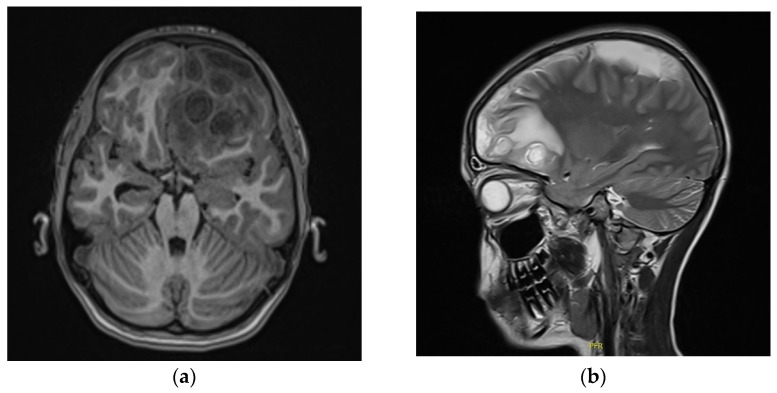
Magnetic resonance imaging (MRI) of the head on 28 December 2021 revealed: (**a**,**b**) significant progression of brain abscesses with edema and mass effect leading to displacement of midline structures at the right side.

At the end of December 2021, due to a significant deterioration of the general condition (i.e., the patient was conscious, but lethargic, with headaches, vomiting, and slight weakness in the right limbs 4+/5 on the Lovett’s scale of muscle strength), an urgent MRI examination of the head was performed. The MRI demonstrated significant progression of brain abscesses with edema and mass effect, leading to displacement of midline structures at the right side (Figure 2a,b). Partial recanalization of the superior sagittal sinus was described. Due to subdural empyema, mainly in the left frontoparietal area, neurosurgical treatment with left parietal craniotomy was applied. The operation was uneventful. Collected during the procedure purulent content was gathered for examination—a negative microbiology culture results were obtained. After the procedure, the boy’s condition remarkably improved, and limb paresis was no longer observed. 

A control MRI of the brain on 10 January 2022 disclosed regression of the previously described changes: size and number of abscesses, size of empyema and sinus inflammation, as well as residual sagittal thrombosis. An MRI of the cervical spine was without pathologies. The control chest X-ray was without obvious infiltrative or inflammatory changes, and complete regression of previous changes was described. MRI of the brain on 27 January 2022 reported further regression of changes in the type of abscesses and empyema and inflammatory infiltration of the frontal lobes (areas of diffusion restriction) with ischemic changes in the upper part cortex; however, re-intensification of inflammatory changes were observed in the paranasal sinuses (ethmoid, frontal, sphenoid). A CT of the sinuses depicted inflammatory masses in the sphenoid sinus and thickening of the mucous membranes of the maxillary, frontal, and ethmoid sinuses. Before being discharged, with the results of the above imaging studies, an ENT re-consultation with an endoscopy examination took place, followed by antibiotic therapy with oral amoxicillin/clavulanic acid for the next 14 days, inhalations with mucoactive agents and nasal steroid therapy with mometasone furoate. The patient was discharged home in good general condition, with a recommendation of scheduled follow-up examinations. 

Follow-up examination on 22 February 2022—laboratory test results were normal, without deviations. Follow-up MRI of the head on 31 August 2022—further regression of the previously described changes with residual thrombotic changes in the superior sagittal sinus. Partial sinus inflammations in the ethmoid and left sphenoid sinus. Neurological state was good.

## 3. Discussion

Typically, it is assumed that pediatric COVID-19 infection has lower risk of hospitalization in comparison to adult patients. Up to 65% of young patients may be asymptomatic, and among the general symptoms, fever and cough are the most common. Less frequently, in atypical cases, anosmia and croup may be observed [13]. Notwithstanding, the available literature shows the multiplicity of SARS-CoV-2 infection complications in this population. They can manifest during isolated COVID-19 infection, after recovery from COVID-19, as well as in the context of multisystem inflammatory syndrome in children (MIS-C). The most reported neurological symptoms in youth are headaches, altered mental status and seizures. When assessing clinical manifestations, cerebrovascular accidents, inflammatory disorders of the central nervous system or muscular weakness are stated. It is possible that neurologic manifestations associated with COVID-19 infection are bound up with different pathogenesis [14,15]; however, a higher incidence was observed in infected boys compared to girls [16].

Our clinical case showed a patient who suffered from acute sinusitis complicated by SARS-CoV-2 co-infection. In the initial stage of the disease, this patient presented typical symptoms of sinus infection in the form of nasal congestion and discharge and headaches. Similar symptoms may be also observed during active SARS-CoV-2 infection, which can lead to misdiagnosis [17]. However, the further course of the disease and the appearance of neurological complications required urgent hospitalization and an investigation into the possible causes of significant deterioration of the patient’s health.

Due to the unclear origin and the negative microbiology culture results, the boy was diagnosed with unspecified encephalitis and myelitis. Several possibilities as predisposing factors should be considered:

- Complication of sinusitis (direct expansion) with contiguous spread;
- Immunodeficiencies caused by SARS-CoV-2 infection with secondary complications;
- Viral respiratory tract infections predisposing to the development of bacterial sinusitis;
- Hematogenous spread from a distant focus (pulmonary origin) caused by COVID-19;
- Opportunistic infection or co-infection following COVID-19 infection.

### 3.1. Sinusitis

Both acute and chronic sinusitis are diseases that affect most people at some point in time. Typically, this disorder is classified depending on the time criterion: acute sinusitis lasting up to 4 weeks, subacute type 4–12 weeks, and chronic infection lasting longer than 12 weeks. Bacterial and fungal etiology most frequently lead to neurological complications in the form of suppurative disease and non-suppurative complications. They can be life-threatening and carry a significant rate of morbidity or long-term sequelae. In case of secondary complications, broad spectrum antibiotics with both aerobic and anaerobic coverage should be started immediately until cultures are performed [18,19]. Otto W et al. [20] assumed that, especially in patients who did not improve after antibiotic therapy, there is a high probability of complications after >1 week of sinusitis symptoms. The most common complication is orbital extension then intracranial extension in the form of suppurative disease (brain and epidural abscess, subdural empyema), and non-suppurative complications (meningitis, cerebritis and/or cerebral venous sinus thrombosis).

Information on viral respiratory tract infections preceding the development of bacterial sinusitis in young children can be found in the literature. Rhinovirus and influenza viruses are mentioned as viruses with the highest complication rates of acute bacterial sinusitis episodes in young children, while girls are more likely to develop complicated upper respiratory tract infection (URI) [21,22]. Plausibly, the rate of sinusitis is associated with severity of the initial URI, which may be related to slower histologic recovery of the mucosa compared with clinical recovery [23]. Meanwhile, a Swedish population-based study [3] analyzed complications caused by acute rhinosinusitis in children 5–18 years old. The incidence of admission was 7.8 per 100.000 children per year, of which severe sinusitis complications were noted in almost 34% of cases. The predominance of boys over girls was observed in higher median age, incidence of admission, complication rate and surgery treatment. Also, the pediatric research conducted in the Netherlands [24] reported a male to female ratio of 2.6:1, whereof 65% of patients had orbital and 31% intracranial complications. Further research is needed to assess whether SARS-CoV-2 infection may also lead to development of bacterial sinusitis in young children.

Other literature reports similar pediatric cases in which patients suffered from sinusitis co-infected with COVID-19, which led to intracranial complications. Reed W. et al. [25] reported a 10-year-old male hospitalized due to progressive periorbital oedema preceded by fever and left-sided frontotemporal headaches. At admission, he tested positive for COVID-19 and his symptoms—acute bacterial sinusitis—were related to the previous contact with his COVID-19-positive father. Due to the unsatisfactory response to intravenous treatment as well as the location and size of the orbital infection, it was decided to perform endoscopic surgery and drainage. Streptococcus pneumoniae was obtained from the collected swabs. Moreover, Blanco et al. [26] reported two cases of pediatric patients with complicated rhinosinusitis and concurrent, recent COVID-19 infection. First, a 15-year-old male patient was hospitalized due to right-sided rhinosinusitis with an epidural collection posterior to the right frontal sinus and superior ophthalmic vein thrombosis. Intraoperative cultures grew coagulase-negative Staphylococcus. The second patient, a 12-year-old male presented a blocked nose and progressive swelling in the right eye. His CT scan presented right-sided rhinosinusitis and subperiosteal abscess. A follow-up after six weeks revealed persistent right-sided rhinosinusitis with opacification of the maxillary, ethmoid, and frontal sinuses. A right-sided endoscopic sinus surgery was performed.

Currently, many reports on fungal sinusitis and/or complications of Mucormycosis connected with SARS-CoV-2 infection are available [27,28,29]; however, very few of them report about bacterial contamination. As the risk factors of opportunistic infections in COVID-19, the authors mention steroid treatment, immunocompromised state, and underlying comorbidities, such as diabetes mellitus and diabetic ketoacidosis, malignancies, or malnutrition. The same may be especially relevant in COVID-19-positive patients [30]. The fungal infection does not seem to be significant in our case because the patient responded well to the received broad spectrum antibacterial treatment. In addition, the boy had no chronic diseases and did not receive steroid treatment prior to the onset of the above symptoms.

### 3.2. Brain Abscesses

The pathophysiology of the appearance of brain abscesses (as well as subdural and extradural empyema) in the pediatric population is various. They are the outcome of [31]:

- Direct expansion of intracranial growth (or infection of contiguous structures) associated with otitis, sinusitis, as well as untreated dental diseases.
- Contiguous spread, which leads to the emergence of retrograde meningitis, cavernous sinus thrombosis, and epidural, subdural, and brain abscess.
- Retrograde spread, as an alternative route of entry, by the intracranial venous system with the valveless venous network. This type is observed in paranasal sinus disease or its acute exacerbation of chronic inflammation.
- Direct spread after head injury or trauma with open fractures.
- Hematogenous spread from a distant focus.

The most frequent abscess localization is in the frontal lobe, then in the parietal and temporal lobes. This localization is influenced by the primary infection site; thus, dental infection as well as frontal or ethmoidal sinusitis have an influence on the abscess location in the frontal lobe. The localization in the parietal or temporal lobes is associated with prior acute otitis media, sphenoidal sinusitis, or mastoiditis. Also, less frequent abscesses of the cerebellum or brainstem are related to hematogenous or otogenic origin [32]. According to the data provided by Szyfter W. et al. [33], sinusitis complications more probably occur concomitantly, and the most frequent abscesses—in the frontal or parietal lobes—were followed by epidural abscess and meningitis. 

The highest probability of intracranial involvement relates to frontal sinusitis which may be related to their most frequent occurrence. Nonetheless, the complication rates for hospitalized patients with various sinusitis reach 3–5% [34,35]. If the source of infection is undetermined, which is more common in children than in adults, an infection is classified as cryptogenic. In the majority of cases in which the symptoms are not immediate, the anticipatory symptoms last for up to two weeks before diagnosis, and its manifestation depends on the occupied space. Among the generalized symptoms of brain abscesses altered consciousness, headaches, vomiting, and fever may be mentioned, while hemiparesis, ataxia, or localized seizure are included in the neurological symptoms. Local symptoms related to infection, such as otalgia, purulent rhinorrhea, or otorrhea, are also less frequent. However, a typical triad of symptoms containing headache, fever, and focal neurologic deficit is observed in up to one-fourth of patients (9–28%) [36,37].

For immunocompetent patients, more than 95% of brain abscesses resulted from bacterial infections and entered the brain through contiguous spread (40–50%) or through hematogenous dissemination (30–40%). Pathogenesis should be established to determine treatment and secondary prevention [38]. The question arises whether a similar pathogenesis should not be considered in patients with COVID-19 infection, where the viral infection leads to fatigue and weakness and propagates the emergence of co-infections or exacerbation of an existing bacterial infection. This question is remains unanswered. Pasquini Z. et al. [39] noticed an increased incidence of bloodstream infections (mainly secondary) in adult patients suffering from SARS-CoV-2. This observation may be explained by several factors, including immune system dysregulations, duration of hospitalization, immunosuppressive treatment, and changes in gut microbiota. Nonetheless, available data reports a relatively small proportion of bacterial co-infection in patients with previous COVID-19 [40,41], which allows us to suspect direct expansion associated with bacterial sinusitis as the origin of abscesses in our patient.

### 3.3. Thrombotic Complications

Increased thrombotic complication rate is mentioned both in acute bacterial sinusitis [22] and during SARS-CoV-2 infection. Therefore, it seems that the coexistence of both diseases may significantly increase this risk, which happened in the case of the presented patient. Anticoagulation therapy with low molecular weight heparin was started immediately, and the dosage was adjusted according to antiXa therapeutic levels. SARS-CoV-2 virus uses an ACE2 receptor to enter the human body. Moreover, those receptors are expressed in the various cells, and their location makes them potential targets for the virus. Replication of the virus leads to dysregulation of inflammation and coagulation, as well as dysregulation of blood pressure, while ACE2 receptors play a significant role in the autoregulation of cerebral vessels and blood flow. This improper regulation causes hypertensive peaks and endothelial pathology which assist the pathogenesis of ischemic and hemorrhagic stroke. Another mechanism leading to coagulation system failure may be a cytokine storm that occurs during COVID-19 infection and leads to inflammatory reactions [42,43]. Coagulation test results changed in 20–50% of patients hospitalized with COVID-19, which is associated with more thrombotic than hemorrhagic events. Observed deterioration of coagulation parameters indicates progression of viral infection and requires increased intensity of care [44]. Pediatric studies report that the incidence of thrombotic events is visibly higher in symptomatic patients hospitalized with COVID-19 or MIS-C, compared to asymptomatic individuals (2.1%, 6.5% and 0.7% respectively). However, thrombotic, or thromboembolic, events remain rare in youth. It is interesting that most cases developed this complication despite the use of thromboprophylaxis, which was also observed in the adult population. Ethnic differences are also noted, with increased prevalence of thrombotic events in African American and Hispanic patients [45,46,47]. It is also suggested that hypercoagulable effects of SARS-CoV-2 may persist for some time following infection [48]. 

### 3.4. Pulmonary Abscess

The authors did not find reported pulmonary complications of COVID-19 infection in the form of lung abscess in children, but some adult cases are available [29,49]. Tissue damage caused by SARS-CoV-2 infection might be the explanation for this find. Hypothetically, the respiratory system could also be the original place from which the infection spread through the bloodstream to the central nervous system. This hypothesis, however, seems unlikely as the boy did not report respiratory symptoms.

Two types of lung abscesses are mentioned: primary (as a result of primary infection) and secondary (caused by other medical conditions, such as extrapulmonary abscesses or pulmonary thromboembolism). 

Nonetheless, we cannot forget about the more common primary lung abscesses caused by aspiration from oral cavity (oropharyngeal secretions such as paradental infection, paranasal sinusitis, or disturbance states of consciousness) [50], which seems probable in this clinical case. Our patient had a screening test for tuberculosis, and the results came back negative. Because of known recent COVID-19 infection and improvement of lung abscess after administration of antibiotics, invasive research was not necessary for the patient. 

### 3.5. Intracranial Complications Differentiation

A ten-year study conducted in the United States [51] reports a declining number of acute rhinosinusitis (ARS) cases in children; however, they also recorded a trend toward the disease appearing in older age with a predominance in boys. Importantly, authors noted an increased number of ARS complications in the form of orbital and intracranial complications. Over a ten-year interval, orbital complication rates increased from 8.9% to 19.3%, while intracranial complication rates changed from 2.2% to 4.3%. The occurrence of both complications simultaneously was seen in 0.5–1% of all ARS discharges. The authors were unable to identify underlying pathogenesis; however, they point to bacterial ARS as the major causative agent [52]. Pott’s puffy tumor is also mentioned as a rare complication of frontal sinusitis. Its clinical presentation varied from forehead swelling or frontal headaches to purulent or non-purulent rhinorrhea. This complication results in a subperiosteal abscess from frontal osteomyelitis and can lead to serious consequences in the form of bacterial meningitis, intracranial abscess, and venous sinus thrombosis [53]. Further investigation in pediatric patients remains difficult as the symptoms of sinus infections, if present, overlap with uncomplicated viral upper respiratory tract infections. The onset of symptoms, such as lethargy, photophobia, neck stiffness, or localized neurological deficits, may direct the suspicion towards an ongoing intracranial process; nevertheless, their absence does not exclude the above process [54].

Of 3707 pediatric patients presented in the systemic review and meta-analysis [55] of neurological manifestation of COVID-19 up to 16.7% had non-specific presentation, such as fatigue, headache, or myalgia. In forty-two cases (1.0%), specific neurological manifestation in the form of seizure, encephalopathy, meningeal signs, cranial nerve involvement, or vision changes occurred. Encephalopathy dominated in this group; however, it resulted not only from a viral complication, but also from complications, such as septic shock or hypoxia. Additionally, seizures and encephalopathy were more common in children with more severe SARS-CoV-2, e.g., in the course of multisystem inflammatory syndrome or Kawasaki-like presentation. Among the symptoms indicating the involvement of the cranial nerves, anosmia should be mentioned since even 85.6% of COVID-19-positive patients will complain of impaired sense of smell. Its appearance indicates the involvement of the cranial nerves and is indicative of neurogenic properties of the virus. Anosmia occurs after the virus binds to the ACE2 receptor and testifies about viral presence in the olfactory bulbs [56]. Relative absence of rhinorrhea (present in 4% of cases) [57] can help differentiate viral etiology from sinusitis. Among central nervous system presentation, Siracusa L. et al. [14] also reported one case of acute disseminated encephalomyelitis and acute cerebrovascular accidents in ten children (four cases of ischemic stroke, three cases of intracerebral hemorrhage, and one case each: a subarachnoid hemorrhage, a multiple diffuse microhemorrhages and a cerebral sinus venous thrombosis). More “typical” intracranial complications in the course of SARS-CoV-2 infection, such as septic venous thrombosis, subdural empyemas, and herniation, were also described in children. The authors see hypercoagulability, immune/endothelial dysfunction, and superinfection as the cause of those complications [58].

The above data indicate that COVID-19 and sinusitis differentiation can be confusing, because presented symptoms may overlap or mimic each other, especially in uncomplicated viral upper respiratory tract infection, which will lead to misdiagnosis. By itself, this viral infection leads to intracranial complications less frequently than sinusitis; however, it may provoke other serious neurological complications in children. Increased risk of complications, especially in patients with bacterial sinusitis co-infected with COVID-19, should prompt physicians to monitor young patients and inform parents about disturbing symptoms and possible complications.

This review has several limitations. The included studies mostly focus on specific neurological symptoms and complications. The number of reports is limited and due to the ongoing pandemic, and many related studies have not yet been published. In order to clarify the relationship between SARS-CoV-2 and sinusitis, further research is required. The next step should be to assess whether SARS-CoV-2 infection may increase the rates of acute bacterial sinusitis episodes, as has been proven for rhinovirus and influenza infections.

## 4. Conclusions

This work introduces a complex issue, as well as numerous similarities between acute sinusitis and COVID-19 infection in relation to the child population. Because the SARS-CoV-2 virus cannot be a direct cause of bacterial infection in the cranial cavity, the main pathology in this patient was likely acute sinusitis, and SARS-CoV-2 co-infection worsened his general and neurological state and thereby his prognosis. 

In conclusion, the available data allow us to deduce that an acute sinus infection that overlaps with SARS-CoV-2 co-infection may lead to more pronounced complications or faster progression and should be included among the factors predisposing to a worse prognosis in pediatric patients, especially since diseases can manifest themselves in the same way. It is assumed that this applies to patients with risk factors for the development of opportunistic infections, the immunocompromised, those with underlying comorbidities, as well as those receiving chronic antibiotic therapy without the desired effect, but our case shows that children without a medical history may also be at risk. Both acute and chronic paranasal sinusitis, especially with the COVID-19 pandemic increasing the risk of superinfection, should be monitored as they have a high possibility of developing intracranial complications, which are life-threatening. Further investigation of the SARS-CoV-2 virus is crucial. It is even more important in children, as they often pass the infection asymptomatically and its complications can lead to loss of health or life.

## Data Availability

Not applicable.
